# Nutrition Knowledge in Oncology Nursing: A Cross‐Sectional Analysis of Care and Practice Implications

**DOI:** 10.1002/nop2.70443

**Published:** 2026-04-08

**Authors:** Giuseppe Alaimo, Stefano Mancin, Sergio Ferrante, Emanuela Morenghi, Sara Morales Palomares, Daniela Cattani, Simone Cosmai, Diego Lopane, Fabio Petrelli, Giovanni Cangelosi, Beatrice Mazzoleni

**Affiliations:** ^1^ Department of Biomedical Sciences Humanitas University Milan Italy; ^2^ IRCCS Humanitas Research Hospital Milan Italy; ^3^ Department of Pharmacy, Health and Nutritional Sciences (DFSSN) University of Calabria Rende Italy; ^4^ School of Pharmacy Polo Medicina Sperimentale e Sanità Pubblica Camerino Italy; ^5^ Unit of Diabetology, Asur Marche Fermo Italy

**Keywords:** cancer, knowledge, nurse, nutrition, oncology care, self‐assessment

## Abstract

**Background:**

Cancer‐related malnutrition affects 40%–90% of oncology patients and contributes to increased treatment toxicity and mortality. Nurses play a pivotal role in early nutritional screening and intervention, yet their nutritional competencies remain insufficiently investigated, particularly in specialised oncology settings.

**Aim:**

To evaluate the nutritional competencies of nurses, considering both their general nutritional knowledge and their specific expertise related to nursing practice.

**Design:**

Cross‐sectional study.

**Methods:**

A total of 91 oncology nurses from Northern Italy participated, completing a validated nutritional knowledge questionnaire and a self‐assessment of their competencies. Associations between demographic factors and knowledge scores were analysed.

**Results:**

The mean knowledge score was 20.0 ± 1.9 out of 30, indicating moderate understanding. Nurses aged 51–60 and those with over 10 years of experience scored significantly higher (*p* < 0.05). No significant association was found between education level and knowledge scores. Nurses reported low confidence in using malnutrition screening tools, and self‐assessed competencies did not correlate with actual knowledge.

**Conclusions:**

Oncology nurses possess moderate nutritional knowledge, with professional experience contributing more than formal education. Implementing comprehensive nutrition education at the undergraduate level, continuous professional development and fostering interprofessional collaboration are essential to enhance the quality of care and improve patient outcomes in oncology settings.

**Relevance to Clinical Practice:**

The findings highlight the need for enhanced nutritional education for oncology nurses to improve their confidence and competence in nutritional care. This is crucial for providing optimal care and improving patient outcomes in oncology settings.

**Patient and Public Contribution:**

The study underscores the importance of involving oncology nurses in continuous education and interprofessional collaboration, ultimately benefiting patients through better management of nutrition‐related issues in cancer care.

## 1 Background

Cancer‐induced malnutrition arises from a combination of metabolic disturbances and appetite loss, driven either by the tumour itself or by associated treatments. This condition severely impacts clinical outcomes and increases the mortality risk in cancer patients (Muscaritoli et al. 2021). Nutritional deficits are linked to a reduced tolerance of cancer therapies, resulting in higher toxicity, lower adherence, decreased treatment response, increased complications, less effective postoperative recovery and longer hospital stays (Arrieta et al. 2015; Bozzetti 2017). Cancer patients often face not only physical limitations but also significant declines in quality of life, spanning psychological, cognitive, social and emotional aspects (Bossi et al. 2022). In 2022, GLOBOCAN estimated nearly 20 million new cancer cases and 9.7 million cancer deaths worldwide. The global cancer burden is projected to reach 35 million cases by 2050 (Bray et al. 2024). Cancer‐related malnutrition occurs frequently, with a prevalence ranging from 40% to 90%, which may be caused by the tumour itself, side effects of treatment and changes in the patient's physical condition (Li et al. 2024). Malnutrition adversely affects quality of life and increases treatment‐related toxicities, with estimates suggesting that 10%–20% of cancer patients die from complications of malnutrition rather than from the tumour itself, yet cancer‐related malnutrition remains widely unrecognised, underestimated and undertreated in clinical practice worldwide (Muscaritoli et al. 2021). The negative energy balance and skeletal muscle loss observed in cancer patients is driven by a combination of reduced food intake and metabolic derangements (e.g., elevated resting metabolic rate, insulin resistance, lipolysis and proteolysis which aggravate weight loss and are provoked by systemic inflammation and catabolic factors) which may be host‐ or tumour‐derived, can only be partially reversed by conventional nutritional support due to the persistence of these metabolic changes (Arends, Bachmann, et al. 2017; Arends, Baracos, et al. 2017). Screening is key to identifying the risk of malnutrition, if nutrition risk is not assessed at the first oncologic visit, nutritional deficiency will be missed in half the patients, and appropriate measures to counteract will not be timely implemented (Arends, Bachmann, et al. 2017; Arends, Baracos, et al. 2017). Independently of the selected criteria or parameters, nutritional status should be regarded as a dynamic concept, especially in oncology, with nutritional screening tests administered early and periodically repeated, preferably by nurses, throughout the patient's journey at each outpatient visit and within 48 h of hospital admission (Caccialanza et al. 2022). On average, malnourished patients stay in hospital longer, approximately 3 days longer, than well‐nourished ones, and, as a result, cost substantially more, per hospital stay (Curtis et al. 2017). Nurse‐led empowerment‐based education has shown significant positive effects by enhancing patients' knowledge of malnutrition management and reducing the frequency of outpatient visits, thus promoting greater autonomy and optimising healthcare resource utilisation (Tuominen et al. 2023).

Nurses play a vital role in nutrition screening and can implement positive behavioural changes to improve the effectiveness of nutritional interventions, with collaborative input from other healthcare professionals further enhancing nutritional outcomes, especially as nurses are daily providers of care and bear responsibility for nutritional interventions (Mancin, Sguanci, et al. 2024; Mancin, Soekeland, et al. 2024). A previous study (Richards et al. 2020) demonstrated that early nutritional counselling in cancer patients significantly improved body weight, nutritional status and quality of life during treatment. Individualised, weekly nutrition counselling for patients with head and neck cancer has been shown to enhance nutritional status, improve quality of life and reduce unplanned hospitalisations (Tunzi et al. 2022). In a separate study, dietary counselling was found to improve treatment adherence and reduce post‐treatment complications in cancer patients (Hamaker et al. 2021). Prehabilitation that includes nutritional counselling has proven effective in maintaining caloric intake, preventing malnutrition and enhancing quality of life in patients undergoing treatment for head and neck cancer (De Pasquale et al. 2023). A structured counselling intervention, encompassing both nursing and nutritional support, has proven to be essential in significantly improving discharge management, controlling treatment side effects and promoting better nutritional behaviours in colorectal cancer patients (Reiter et al. 2021). Nurses, through nurse‐led dietary interventions, may help improve the intake of fruits, vegetables and energy among cancer patients (Gan et al. 2022).

In Italy, there is no national legislation that mandates systematic nutritional screening for patients in hospital or home care settings. However, the Lombardy Region has introduced a regional decree that makes nutritional screening mandatory for all patients receiving care in healthcare and social care facilities, as well as for those in home care. This regulation stipulates the use of validated tools such as the ‘Malnutrition Universal Screening Tool’ (MUST), the ‘Nutritional Risk Screening 2002’ (NRS‐2002) and ‘StrongKids’ for paediatric patients to ensure the early identification of individuals at risk of malnutrition (Regione Lombardia 2024).

Despite the recognised importance of nutritional care in oncology, significant gaps persist in nursing education and competency regarding clinical nutrition. A systematic review by Zeldman and Andrade (2020) revealed inconsistencies in nutritional knowledge among healthcare professionals, highlighting the need for targeted assessment and training interventions. In Italy, while the Lombardy Region has mandated nutritional screening using validated tools (MUST, NRS‐2002) for all patients in healthcare facilities (Regione Lombardia 2024), compliance and nursing staff preparedness remain understudied. Previous studies have documented heterogeneity in how nutrition content is integrated into nursing curricula (Yuste Muñoz et al. 2023), with variability in teaching methods, faculty expertise and practical training opportunities. This inconsistency may contribute to disparities in nurses' ability to identify and manage malnutrition risk, particularly in complex oncology populations where nutritional deficits are highly prevalent and clinically consequential. Furthermore, research suggests a potential discrepancy between nurses' self‐perceived competencies and their actual proficiency in clinical nutrition (Gbareen et al. 2021). Such misalignment can have significant implications for patient care, as overestimation of skills may lead to underutilisation of evidence‐based screening tools or failure to recognise nutritional risk, while underestimation may result in missed opportunities for effective intervention. To date, no studies have systematically evaluated both objective nutritional knowledge and self‐assessed competencies among oncology nurses in the Italian context, where regional mandates for nutritional screening create specific expectations for nursing practice. Understanding this dual dimension—actual knowledge and perceived competence—is essential to design targeted educational interventions that address both skill deficits and metacognitive awareness. This study addresses this gap by assessing nutritional competencies among oncology nurses using a validated knowledge questionnaire and a self‐assessment instrument, enabling identification of specific areas requiring educational reinforcement and evaluation of congruence between perceived and actual proficiency.

### 1.1 Theoretical Framework

This study is grounded in Donabedian's Structure‐Process‐Outcome framework (Donabedian 1988), which conceptualises quality of care across three dimensions: structural attributes (e.g., nursing education, institutional resources), care processes (e.g., nutritional screening, patient counselling) and patient outcomes (e.g., nutritional status, treatment tolerance). In the context of oncology nursing, nutritional knowledge and competencies represent structural components that directly influence care processes specifically, nurses' ability to identify malnutrition risk, implement evidence‐based interventions and collaborate interprofessionally to optimise patient nutritional status.

By assessing both objective knowledge (a structural element) and self‐perceived competencies (a process‐related attribute reflecting readiness to act), this study evaluates foundational prerequisites for effective nutritional care delivery. The framework posits that deficits in either dimension—knowledge gaps or metacognitive misalignment—may compromise care processes and, ultimately, patient outcomes. This lens informs the study design and guides interpretation of findings regarding educational and organisational interventions required to enhance nursing‐led nutritional care in oncology settings.

### 1.2 Aims and Objectives

To evaluate the nutritional competencies of oncology nurses, assessing both their general nutritional knowledge and their self‐perceived expertise related to clinical nursing practice. The study aims to quantify the level of nutritional knowledge among oncology nurses using a validated assessment tool, and to identify demographic and professional factors—including age, years of experience and educational background—associated with nutritional knowledge scores. Additionally, the research seeks to evaluate nurses' self‐assessed confidence in key competencies related to clinical nutrition, encompassing evidence retrieval, malnutrition screening, nutritional support administration and patient education. Finally, the study examines the correlation between self‐assessed competencies and objective knowledge scores to identify potential discrepancies between perceived and actual proficiency.

## 2 Methods

### 2.1 Study Design

This study was designed as a cross‐sectional observational investigation and was conducted in accordance with the STROBE guidelines to ensure the clarity and transparency of reporting observational research (Vandenbroucke et al. 2007) (Supporting Information). Nurses were recruited, and their general nutritional knowledge was assessed using a validated questionnaire developed by Da Vico et al. (2015). Additionally, a self‐assessment questionnaire was administered to evaluate participants' perceived knowledge and competencies specifically in clinical nutrition. This instrument was designed to assess self‐reported proficiency and confidence in skills directly applicable to clinical practice, with a particular focus on domains essential to effective nutritional management and patient care. A quantitative research design was chosen as the most appropriate approach for this study. According to Cathala and Moorley (2018), quantitative methods are optimal when the research aim is to measure, quantify and generalise findings across a defined population. Given our objectives—to assess the magnitude of nutritional knowledge deficits, identify statistically significant associations between demographic variables and competency scores, and enable comparison with findings from other healthcare settings—a survey‐based quantitative approach provided the necessary rigour and standardisation. This design allows for objective measurement of knowledge using a validated instrument with established psychometric properties, facilitating replicability and comparison with existing literature.

The study adhered to the guidelines of the Declaration of Helsinki and received approval from the Institutional Review Board under the approval code NUT 23/04. All participants provided informed consent prior to their inclusion in the study.

### 2.2 Context of the Research

The study was conducted at a tertiary research hospital located in Northern Italy, known for its status as a scientific research institution and its expertise in advanced medical care, where the nursing staff comprises individuals from various regions across the country, each with potentially diverse academic backgrounds from different universities. This variation in educational pathways may contribute to differences in the nurses' level of nutritional knowledge.

### 2.3 Participants and Criteria

The study enrolled a sample of 159 participants. Inclusion criteria included nurses who provided care to patients with oncological diseases. Exclusion criteria encompassed nurses who, despite working with oncological patients, were ineligible due to short‐term employment or the inability to perform nutritional counselling, such as those assigned to operating rooms, emergency departments or intensive care units, as well as nurses who declined to have their personal data processed.

A purposive sampling strategy was employed for this study. This non‐probabilistic approach was justified by the study's focus on a specialised population—oncology nurses working in direct patient care roles where nutritional screening and intervention are clinically relevant. As highlighted by Shorten and Moorley (2014), purposive sampling is appropriate when the research question requires accessing participants with specific characteristics or expertise that are not uniformly distributed in the general population. Given the exploratory nature of this study and the need to target nurses actively engaged in oncology care, purposive sampling enabled efficient recruitment while ensuring that participants met inclusion criteria critical to addressing the research objectives. While this approach limits statistical generalisability, it enhances the relevance and applicability of findings to the target population.

Participants received a link to the questionnaire through their institutional email, directing them to a Google Form. The questionnaire remained accessible for a 2‐week period from the time of receipt, allowing participants ample time to complete the survey.

### 2.4 Evaluation Tool Used and Data Collection

In this study, a previously validated Italian questionnaire (Da Vico et al. 2015) was employed to assess the level of understanding regarding fundamental aspects of clinical nutrition. The questionnaire was originally developed by Moynihan et al. (2007) and is an abbreviated version of an earlier tool created by Parmenter and Wardle (1999). It is designed to be a straightforward, quick‐to‐administer instrument, making it well‐suited for clinical use. However, the original version was developed within an Anglo‐Saxon cultural framework, which differs significantly from the Italian context, particularly with respect to dietary habits. Therefore, in a previous study (Da Vico et al. 2015), the tool was both translated and culturally adapted to reflect the most consumed foods in Italy, and the Italian version was subsequently validated. The instrument has demonstrated appropriate psychometric properties for its application in clinical research. It comprises 15 questions in total: the first section (questions 1–10) addresses basic nutritional knowledge, while the second section (questions 11a–11d) focuses on specific nutritional knowledge related to diseases caused by poor dietary practices, reflecting aspects of clinical nutrition. Correct answers are assigned a score of 1, while incorrect responses are scored 2. For the multiple‐choice questions (questions 4 and 6), each correct answer is awarded 0.1 points, and each incorrect answer receives 0.2 points. The lowest possible score, indicating the best performance, is 15 points, and the highest possible score, reflecting the poorest performance, is 30 points. Questions 11a through 11d allow for a qualitative assessment of the responses, providing further insight into specific areas of nutritional knowledge. Although this qualitative data does not contribute to the overall questionnaire score, it is used for a descriptive analysis to enhance the understanding of the participants' knowledge. No formal pilot study was conducted prior to full‐scale data collection. This decision was based on the following considerations: (1) the questionnaire had been previously validated in the Italian context (Da Vico et al. 2015), with established psychometric properties demonstrating reliability and construct validity; (2) the instrument had been successfully employed in prior studies with Italian healthcare professionals, providing evidence of cultural and linguistic appropriateness; and (3) institutional ethical review timelines and resource constraints precluded pilot testing within the project's feasibility parameters. While pilot testing may have identified minor contextual adaptations, the use of a validated instrument mitigated concerns regarding comprehensibility and response burden.

### 2.5 Self‐Assessment Questionnaire

An additional self‐assessment questionnaire was developed specifically for this study to evaluate participants' perceived knowledge and competencies in clinical nutrition. The instrument was designed based on a review of competency frameworks for nursing nutritional care (Boeykens and Van Hecke 2018; Mancin, Soekeland, et al. 2024) and regional regulatory requirements for nutritional screening (Regione Lombardia 2024). Six core competency domains were identified through expert consultation (*n* = 3 senior oncology nurses, *n* = 1 clinical nutritionist) and aligned with evidence‐based practice expectations: (1) evidence retrieval and critical appraisal, (2) malnutrition risk identification using validated tools, (3) administration of oral and artificial nutritional support, (4) patient and caregiver education, (5) interprofessional communication and collaboration, and (6) awareness of regulatory mandates for nutritional screening. Items were rated on a 5‐point Likert scale (1 = ‘Not at all confident’ to 5 = ‘Extremely confident’), allowing for granular self‐assessment of perceived competence. Given the exploratory nature of this study and the instrument's use as a descriptive complement to the validated knowledge questionnaire, formal psychometric validation (e.g., test–retest reliability, factor analysis) was not conducted. A formal pilot test of the Self‐Assessment Questionnaire was not conducted prior to full deployment. This decision was based on three considerations: (1) the primary validated nutritional knowledge questionnaire (Da Vico et al. 2015) had established psychometric properties in Italian healthcare populations, requiring no additional piloting; (2) the Self‐Assessment Questionnaire was designed as an exploratory, descriptive complement rather than a primary outcome measure; and (3) time and resource constraints associated with the single‐site study scope precluded comprehensive pilot testing. To ensure face validity and item clarity, the Self‐Assessment instrument underwent expert review by the consultation panel (*n* = 3 senior oncology nurses, *n* = 1 clinical nutritionist), and verbal confirmation of item comprehensibility was obtained from two additional oncology nurses during instrument refinement. These procedures provided quality assurance for content appropriateness and readability, though they do not substitute for formal pilot testing with test–retest reliability assessment. Ethnicity was not collected as demographic variables in this study. This decision reflects three considerations: (1) the study was conducted in a single Northern Italian institution with a predominantly homogeneous nursing workforce, resulting in minimal ethnic variability that would preclude meaningful statistical analysis; (2) alignment with GDPR (General Data Protection Regulation) principles guiding minimisation of sensitive personal data collection unless directly necessary for research objectives; and (3) absence of established theoretical framework or empirical evidence suggesting race/ethnicity as predictors of nutritional knowledge in the Italian nursing context, unlike well‐documented associations with age, professional experience and educational attainment documented in international literature.

### 2.6 Data Collection

Following ethical approval (CLI_RIC_20), eligible participants received an email invitation containing a hyperlink to a secure Google Forms survey. The email explained the study purpose, voluntary nature of participation, confidentiality protections and estimated completion time (approximately 15 min). Participants provided electronic informed consent prior to accessing the questionnaire by clicking an affirmative checkbox after reviewing study information. Data were automatically stored in the Google Forms database with restricted access limited to the principal investigator. Upon completion of data collection, responses were exported to STATA 18 for analysis. The study adhered to the Declaration of Helsinki principles. Participation was voluntary, and nurses were informed that non‐participation would not affect their employment or professional standing. No incentives were provided for survey completion.

### 2.7 Data Analysis

All statistical analyses were conducted using STATA 18 (StataCorp, College Station, TX, USA).

Demographic and clinical characteristics were summarised using frequencies and percentages for categorical variables (gender, age group, education level, years of experience) and means with standard deviations (SD) for continuous variables (knowledge scores, self‐assessment scores).

Prior to analysis, data were inspected for completeness, outliers and data entry errors. Range checks confirmed that all knowledge scores fell within the instrument's theoretical minimum (15) and maximum (30). No univariate outliers (defined as values > 3 SD from the mean) were identified. Three respondents with incomplete demographic data were excluded listwise from regression analyses.

Normality of continuous variables was assessed using the Shapiro–Wilk test and visual inspection of histograms and *Q*–*Q* plots. Knowledge scores were approximately normally distributed (Shapiro–Wilk *W* = 0.982, *p* = 0.214), supporting the use of parametric tests. For variables violating normality assumptions, non‐parametric alternatives (Mann–Whitney *U*, Kruskal–Wallis) were considered but ultimately not required given sample robustness. Associations between demographic predictors (age group, years of experience, education level, gender) and nutritional knowledge scores were evaluated using multivariable linear regression. Coefficients (*β*), 95% confidence intervals (CI) and *p*‐values were reported. Model assumptions—linearity, independence of errors, homoscedasticity and absence of multicollinearity—were verified using residual plots and variance inflation factors (VIF < 2 for all predictors). Linear regression was chosen over *t*‐tests or ANOVA to allow simultaneous adjustment for multiple covariates and to quantify the independent effect of each predictor while controlling for confounders. For example, age and experience are often correlated; regression enables partitioning of their independent contributions to knowledge scores. Pearson's correlation coefficient (*r*) was calculated to examine the association between knowledge scores and self‐assessment scores, given that both variables met parametric assumptions. A significance threshold of *p* < 0.05 (two‐tailed) was applied. A two‐tailed alpha level of 0.05 was used for all hypothesis tests. Given the exploratory nature of the study, no correction for multiple comparisons was applied; however, this is acknowledged as a potential source of Type I error inflation. No post‐hoc pairwise comparisons (e.g., Tukey HSD) were conducted following regression analysis. The regression framework already provides specific contrast estimates (coefficients) for each predictor level relative to the reference category, rendering traditional post‐hoc tests redundant. This methodological choice is consistent with recommendations for regression‐based analyses (Gelman and Hill 2006) but is noted as a limitation for readers interested in all possible between‐group differences.

## 3 Result

### 3.1 Characteristics of the Participants

Out of the 159 nurses eligible for participation, 94 completed the questionnaire, resulting in a response rate of 59.49%. Three nurses declined to participate, and their data were excluded from the analysis. The sample was predominantly female, comprising 79.12% of the respondents, while 19.78% were male. The largest proportion of respondents belonged to the 22–30 age group, accounting for 45.05% of the total sample (*n* = 41). The educational qualification most frequently reported was a Bachelor's degree, held by 74.73% of the participants (*n* = 68), representing the highest proportion of the sample. In terms of professional experience, the distribution was approximately equal across the different categories, with no category representing a significantly higher proportion of the sample (Table 1).

**TABLE 1:** Sample characteristics.

Variable	Coeff (95% CI)	*p*
**Sex (Male)**	−0.20 (−1.20 to 0.80)	0.696
**Age**		
< 30 years	Ref	—
31–40 years	0.29 (−0.59 to 1.18)	0.511
41–50 years	−0.82 (−1.99 to 0.34)	0.163
51–60 years	−1.81 (−3.30 to −0.31)	0.019
> 61 years	0.12 (−3.58 to 3.82)	0.948
**Years of work experience**		
< 2 years	Ref	—
2–5 years	−0.44 (−1.65 to 0.78)	0.477
6–10 years	−0.75 (−1.99 to 0.48)	0.229
> 10 years	−1.26 (−2.45 to −0.07)	0.039
**Educational level**		
University diploma	Ref	—
Bachelor's degree	2.19 (0.93 to 3.46)	0.001
Master's degree	1.08 (−1.06 to 3.23)	0.318
First‐level Master	1.48 (−0.16 to 3.12)	0.076

### 3.2 Appraisal of Nutritional Knowledge Tool

Ninety‐one out of the 159 eligible nurses completed the questionnaire, resulting in a response rate of 59.49%. The mean score achieved was 20.0 ± 1.9, with a range of 16.3–24.6. The linear regression analysis identified several statistically significant associations between demographic variables and the nutritional knowledge scores. Gender was not significantly associated with the outcome (β = −0.20, 95% CI: −1.20 to 0.80, *p* = 0.696). Regarding age, nurses in the 51–60 age group demonstrated a statistically significant association compared with the reference group (< 30 years) (β = −1.81, 95% CI: −3.30 to −0.31, *p* = 0.019), while no significant associations were observed for the 31–40 (β = 0.29, 95% CI: −0.59 to 1.18, *p* = 0.511) and 41–50 age groups (β = −0.82, 95% CI: −1.99 to 0.34, *p* = 0.163). Concerning work experience, participants with more than 10 years of experience showed a statistically significant association compared with the reference group (< 2 years) (β = −1.26, 95% CI: −2.45 to −0.07, *p* = 0.039); no significant associations were found for the 2–5 years (β = −0.44, 95% CI: −1.65 to 0.78, *p* = 0.477) and 6–10 years categories (β = −0.75, 95% CI: −1.99 to 0.48, *p* = 0.229). Regarding educational level, Bachelor's degree holders showed a statistically significant association compared with the reference category (University diploma) (β = 2.19, 95% CI: 0.93 to 3.46, *p* = 0.001), while Master's degree (β = 1.08, 95% CI: −1.06 to 3.23, *p* = 0.318) and First‐level Master (β = 1.48, 95% CI: −0.16 to 3.12, *p* = 0.076) did not reach statistical significance. (Table 2).

**TABLE 2:** Associations between demographic variables and the nutritional knowledge scores.

Variable	Coefficient	95% CI	*p*
Gender	−0.20	−1.20 to 0.80	0.696
Age 51–60 vs. < 30	−1.81	−3.30 to −0.31	**0.019**
Experience > 10 years vs. < 2 years	−1.26	−2.45 to −0.07	**0.039**
Educational qualifications	0.12	−0.41 to 0.66	0.645

*Note:* Multivariable linear regression coefficients (*β*) with 95% confidence intervals. Reference categories: gender = female, age = < 30 years, experience = < 2 years. Educational qualifications treated as continuous ordinal variable (1 = Diploma, 2 = Bachelor's, 3 = Master's, 4 = Post‐Bachelor's Master). Negative coefficients indicate lower knowledge scores (better nutritional knowledge). Model adjusted for all variables shown. *n* = 88 (complete‐case analysis). Bold formatting to statistically significant results *p* < 0.05.

Further qualitative inquiries regarding clinical nutrition (11–11d) were included, in which nurses were asked to specify the pathologies could be attributed to an incorrect diet. Low fibre intake was predominantly linked to intestinal disorders (88.89%), followed by impaired glucose metabolism (14.81%) and colon cancer (12.96%). Insufficient fruit and vegetable consumption was strongly associated with vitamin deficiencies (70.27%), intestinal issues (33.78%) and electrolyte imbalances (20.27%). High fat consumption was correlated with cardiovascular diseases (58.65%), weight gain (48.24%) and dyslipidaemia (41.18%). Excessive sugar intake was primarily linked to diabetes or insulin resistance (72.09%), hyperglycaemia (33.72%) and obesity (20.93%). High salt intake was most frequently associated with hypertension (77.01%), renal dysfunction (34.48%) and oedema (11.49%) (Table 3).

**TABLE 3:** Questions relating to clinical nutrition.

Question	Respondents affirming association (%)	Most prevalent response (%)	Second prevalent response (%)	Third prevalent response (%)
Q11	59.34	Intestinal disorders (88.89%)	Altered glucose metabolism (14.81%)	Colon cancer (12.96%)
Q12	81.32	Vitamin deficiencies (70.27%)	Intestinal issues (33.78%)	Electrolyte imbalances (20.27%)
Q13	93.41	Cardiovascular diseases (58.65%)	Weight gain (48.24%)	Blood test abnormalities (41.18%)
Q14	94.51	Diabetes/insulin resistance (72.09%)	Hyperglycaemia (33.72%)	Obesity (20.93%)
Q15	95.60	Hypertension (77.01%)	Renal diseases (34.48%)	Oedema (11.49%)

*Note:* Q11–Q15 are open‐ended questions allowing multiple responses. Percentages in columns 2–4 calculated on respondents who provided at least one answer to that question (denominator varies by question: Q11 *n* = 54, Q12 *n* = 74, Q13 *n* = 85, Q14 *n* = 86, Q15 *n* = 87). Column 1 shows percentage of total sample (*n* = 91) who provided any answer.

### 3.3 Evaluation of Self‐Assessment Questionnaire

Participants showed moderate confidence in their ability to retrieve and understand scientific evidence (mean: 2.76, SD: 0.90) and lower confidence in using validated malnutrition screening tools (mean: 2.57, SD: 1.02). Conversely, higher knowledge levels were reported in administering oral nutritional supplements and artificial nutrition (mean: 3.41, SD: 0.84). Perceived competence in delivering educational interventions and providing regular nutritional counselling varied, with some reporting weaker preparation (mean: 3.03, SD: 0.84). Furthermore, there was limited awareness of the regulation mandating nutritional screening for all patients in healthcare and social care settings, as well as for those in home care (mean: 1.60, SD: 0.94) (Table 4).

**TABLE 4:** Self‐assessment questionnaire.

Question	Mode (*n*)	Mean (%)	SD	Median (*n*)	Percentage distribution (score 1–5)
What is your level of knowledge regarding the retrieval and understanding of scientific evidence in clinical nutrition?	3.0	2.76	0.9	3.0	1: 7.69% 2: 30.77% 3: 40.66% 4: 19.78% 5: 1.10%
How prepared do you feel in identifying the risk of malnutrition using validated screening tools such as MUST, MNA or NRS‐2002?	3.0	2.57	1.02	3.0	1: 18.68% 2: 26.37% 3: 34.07% 4: 20.88% 5: 0.00%
What is your level of knowledge regarding the administration of oral nutritional supplements and artificial nutrition (enteral/parenteral)?	4.0	3.41	0.84	3.0	1: 3.30% 2: 7.69% 3: 39.56% 4: 43.96% 5: 5.49%
What is your level of competence in conducting nutritional education interventions for patients and/or caregivers?	3.0	3.03	0.84	3.0	1: 4.40% 2: 18.68% 3: 47.25% 4: 28.57% 5: 1.10%
How familiar are you with the recent regional regulation that mandates nutritional screening for all patients in healthcare and social care facilities, as well as for those receiving home care?	1.0	1.6	0.94	1.0	1: 61.54% 2: 24.18% 3: 8.79% 4: 3.30% 5: 2.20%
Do you regularly conduct nutritional education for patients and/or caregivers as part of your daily practice?	3.0	2.76	0.9	3.0	1: 7.69% 2: 30.77% 3: 40.66% 4: 19.78% 5: 1.10%

*Note:* Response scale: 1 = ‘not at all confident’, 2 = ‘slightly confident’, 3 = ‘moderately confident’, 4 = ‘very confident’, 5 = ‘extremely confident’. Scores presented as mean ± SD with full distribution showing percentage endorsing each level.

Abbreviation: SD, standard deviation.

From the analysis of the obtained results, no significant correlation emerged between the scores of the two questionnaires. Higher self‐assessment scores were not associated with superior knowledge of basic nutritional concepts. This suggests a potential discrepancy between the participants' self‐perceived competence and their actual proficiency in clinical nutrition.

Formal hypothesis testing was not conducted for self‐assessment data due to the instrument's descriptive, non‐validated nature and the study's exploratory objectives. Unlike the nutritional knowledge questionnaire, which has established psychometric properties enabling robust statistical inference, the self‐assessment tool was developed specifically for this study without prior validation. Consequently, self‐assessment scores were treated as descriptive indicators of perceived competence rather than as outcome variables suitable for confirmatory hypothesis testing. This methodological decision aligns with recommendations for pilot or exploratory instruments (Boateng et al. 2018) and is acknowledged as a study limitation necessitating future validation work.

## 4 Discussion

This study aimed to evaluate the nutritional knowledge of nurses, focusing on both their general nutritional knowledge and specific expertise related to nursing practice in an oncology setting. The study demonstrated that the overall nutritional knowledge among participating nurses was moderate, with a mean score of 20.0 ± 1.9 on the appraisal tool. Notably, nurses aged between 51 and 60 years and those with more than 10 years of professional experience exhibited significantly better nutritional knowledge compared to their younger and less experienced counterparts. Conversely, the level of formal education did not show a significant association with nutritional knowledge scores, which is consistent with findings from Zeldman and Andrade (2020), who highlighted that formal education alone often does not suffice in developing specialised competencies in clinical nutrition, underscoring the importance of ongoing professional development. The self‐assessment questionnaire revealed that while nurses felt moderately confident in certain areas of clinical nutrition, such as administering nutritional support, they had lower confidence in utilising validated malnutrition screening tools and were largely unaware of regional regulations mandating nutritional screening. Importantly, there was no significant correlation between the nurses' self‐assessed competencies and their actual knowledge, indicating a potential mismatch between perception and reality. Similar results were reported by Gbareen et al. (2021), who found that despite nurses' belief in their abilities to assess patient nutrition, there was often a gap between self‐perceived and actual competencies, particularly with respect to validated screening tools additionally, nurses often perceive nutritional assessments as lower priority tasks, primarily due to insufficient training and heavy workloads. Participants reported moderate confidence in retrieving and understanding scientific evidence related to clinical nutrition but expressed lower confidence in utilising validated malnutrition screening tools such as MUST, MNA or NRS‐2002. Given that early identification of malnutrition risk is crucial for implementing timely interventions, the lack of familiarity with screening tools is a significant gap in practice (Caccialanza et al. 2022).

The qualitative analysis of responses regarding diseases associated with poor dietary practices showed that nurses could generally identify the correct associations, such as linking high fat intake with cardiovascular diseases and excessive sugar consumption with diabetes.

However, the depth of understanding and the ability to apply this knowledge in clinical scenarios were not assessed, pointing to a potential area for further investigation. The discrepancy between self‐perceived competencies and actual knowledge is particularly noteworthy. Similar patterns have been reported in other healthcare domains, where professionals may overestimate or underestimate their abilities (Dunning et al. 2004). This misalignment can have significant implications for patient care, as it may lead to underutilisation of nutritional interventions or failure to recognise malnutrition risks. Integrating nutrition education across all levels of nursing curricula through a collaborative model involving dietitians and nurse educators has been shown to significantly improve nursing students' competencies in providing nutrition‐focused care (Shea et al. 2021). This interprofessional approach supports the current study's emphasis on the importance of structured and multidisciplinary training interventions. Significant heterogeneity exists across nursing programmes in how nutrition content is structured and delivered, ranging from traditional lectures to hands‐on learning experiences like case studies, clinical simulations and practical assignments (Yuste Muñoz et al. 2023). This disparity extends to the faculty responsible for teaching nutrition, with some programmes relying on dietitians while others entrusting it to nurses or other professionals. Such inconsistencies in teaching methods and faculty expertise underscore a critical gap that could affect the preparedness of nurses to manage nutrition‐related issues in clinical settings.

Studies across various healthcare settings have demonstrated that nurses with advanced expertise in nutrition significantly contribute to improved patient outcomes, especially in complex fields like oncology. The advanced skill sets outlined by Mancin, Soekeland, et al. (2024) and Boeykens and Van Hecke (2018) highlight the necessity of integrating evidence‐based nutritional interventions into clinical practice, promoting interprofessional collaboration and incorporating rigorous nutritional training within nursing education programmes. Such specialised competencies not only elevate the standard of patient care but also empower nurses to proactively address intricate nutritional challenges, including cancer‐associated malnutrition and cachexia.

### 4.1 Limitations

Several limitations should be acknowledged. The study's response rate was 59.49%, which may introduce response bias. The cross‐sectional design limits the ability to infer causality between variables. The study was conducted in a single institution, which may limit the generalisability of the results to other regions or healthcare settings with different resources and patient populations.

### 4.2 Implications for Practice

The findings highlight a need for targeted educational interventions to enhance nurses' nutritional competencies, particularly in oncology settings where patients are at high risk of malnutrition.

The moderate level of nutritional knowledge underscores the need for enhanced educational initiatives targeting nurses, especially those early in their careers. Incorporating comprehensive clinical nutrition training into both undergraduate nursing programmes and continuing professional development courses could bridge the identified knowledge gaps.

Given the pivotal role nurses play in patient care, empowering them with robust nutritional knowledge and skills can lead to better patient outcomes, including improved quality of life, reduced treatment complications and decreased healthcare costs due to shorter hospital stays (Curtis et al. 2017; Tuominen et al. 2023).

## 5 Conclusion

Implementing comprehensive nutrition education from the undergraduate level provides nurses with foundational knowledge essential for effective clinical practice, enhancing their ability to integrate nutritional assessment and intervention into patient care. This foundational training, coupled with continuous professional development, helps ensure that nurses remain proficient in evidence‐based practices and are better prepared to identify and manage patients at nutritional risk across diverse clinical settings. Furthermore, fostering interprofessional collaboration among nurses, doctors and dietitians promotes a cohesive, multidisciplinary approach to patient care. This integrated model not only improves patient outcomes in oncology but also supports personalised care plans, reduces complications and enhances overall patient quality of life, particularly in complex cases where nutrition plays a critical role in treatment and recovery.

## Author Contributions

**Giuseppe Alaimo:** conceptualisation, methodology, investigation, data curation, writing – original draft preparation, writing – review and editing, visualisation. **Stefano Mancin:** methodology, supervision, writing – review and editing, visualisation. **Sergio Ferrante:** writing – review and editing, visualisation. **Emanuela Morenghi:** formal analysis, validation, writing – review and editing, visualisation. **Sara Morales Palomares:** writing – review and editing, visualisation. **Daniela Cattani:** writing – review and editing, visualisation. **Simone Cosmai:** writing – review and editing, visualisation. **Diego Lopane:** writing – review and editing, visualisation. **Fabio Petrelli:** writing – review and editing, visualisation. **Giovanni Cangelosi:** writing – review and editing, visualisation, supervision. **Beatrice Mazzoleni:** writing – review and editing, visualisation, supervision. Giuseppe Alaimo and Stefano Mancin provided an equal contribution as first author in drafting the manuscript. Giovanni Cangelosi and Beatrice Mazzoleni provided an equal contribution as last author in drafting the manuscript. All authors read and approved the final manuscript.

## Funding

The publication fee for this work was covered by the Italian Ministry of Health's ‘Ricerca Corrente’ funding to the IRCCS Humanitas Research Hospital.

## Ethics Statement

The study adhered to the guidelines of the Declaration of Helsinki and received approval from the Institutional Review Board under the approval code NUT 23/04. All participants provided informed consent prior to their inclusion in the study.

## Conflicts of Interest

The authors declare no conflicts of interest.

## Supporting information


Data S1.

## Data Availability

The data that support the findings of this study are available in the Supporting Information of this article.
